# Imagine, and you will find – Lack of attentional guidance through visual imagery in aphantasics

**DOI:** 10.3758/s13414-021-02307-z

**Published:** 2021-04-20

**Authors:** Merlin Monzel, Kristof Keidel, Martin Reuter

**Affiliations:** 1 Personality Psychology and Biological Psychology, Department of Psychology, University of Bonn, Kaiser-Karl-Ring 9, 53111 Bonn, Germany; 2 Center for Economics and Neuroscience (CENs), Laboratory of Neurogenetics, University of Bonn, Bonn, Germany

**Keywords:** Aphantasia, Visual imagery, Visual attention, Attentional guidance, Visual search, Imagery debate

## Abstract

Aphantasia is the condition of reduced or absent voluntary imagery. So far, behavioural differences between aphantasics and non-aphantasics have hardly been studied as the base rate of those affected is quite low. The aim of the study was to examine if attentional guidance in aphantasics is impaired by their lack of visual imagery. In two visual search tasks, an already established one by Moriya (*Attention, Perception, & Psychophysics*, *80*(5), 1127-1142, 2018) and a newly developed one, we examined whether aphantasics are primed less by their visual imagery than non-aphantasics. The sample in Study 1 consisted of 531 and the sample in Study 2 consisted of 325 age-matched pairs of aphantasics and non-aphantasics. Moriya’s Task was not capable of showing the expected effect, whereas the new developed task was. These results could mainly be attributed to different task characteristics. Therefore, a lack of attentional guidance through visual imagery in aphantasics can be assumed and interpreted as new evidence in the imagery debate, showing that mental images actually influence information processing and are not merely epiphenomena of propositional processing.

**Supplementary Information:**

The online version contains supplementary material available at 10.3758/s13414-021-02307-z.

## Introduction

Visual imagery is defined as the ability to create mental representations of stimuli that are similar to visual perceptions (Block, 1983; Dennet, 1978; cf. Winlove et al., 2018), even though the corresponding stimuli are not within the visual field of the imagers (Finke, 1989; Richardson, 2013). In 2015, Zeman et al. coined the term “aphantasia”, defined as a state of reduced or absent voluntary imagery, thus for the first time systematically drawing attention to the fact that not everyone has visual imagery. Those affected are not able to deliberately create mental images, for example when reading books or thinking of close relatives. Initial estimates quantify the amount of people affected at 2–3% of the world population (Faw, 2009; Zeman et al., 2020).

Aphantasia provides a novel approach to the imagery debate between Kosslyn (1981, 2005) and Pylyshyn (1973, 2002, 2003), which has been going on since the 1970s. While both sides acknowledge the existence of mental images, Pylyshyn describes it as an epiphenomenon of propositional processing, which arises as a result of the process but does not influence the process itself. On the other hand, Kosslyn (1975, 1976, 1978), Kosslyn et al. (1978), Kosslyn and Alper (1977), and Shepard and Metzler (1971) presented a series of behavioural experiments to prove that mental images do indeed functionally influence information processing. For example, Shepard and Metzler (1971) showed that the angle of rotation in a visual rotation task influences the response time, which might not be the case in propositional processing. Pylyshyn (1981) countered this argument by claiming that these results were based on tacit knowledge of the participants, that is, the participants implicitly assumed that they processed images, whereupon they adjusted their response times to this assumption. However, with the existence of aphantasics, the imagery debate might now be resolved, because if Kosslyn's theory of image-dependent processing is valid, behavioural differences between aphantasics and non-aphantasics will become apparent, since the former cannot use mental images in information processing. On the other hand, if Pylyshyn's hypothesis is valid, no differences should occur.

However, as a prerequisite for drawing conclusions regarding the imagery debate, it has to be shown that aphantasics actually do not have visual imagery and that behavioural differences do not result from a lack of effort on the part of aphantasics to create mental images, since they assume that this is not possible anyway. Evidence for this comes from neuroscientific research. Pearson (2019) developed the *Reverse Hierarchy Model*, which describes visual imagery as the reverse neural process of visual perception. While visual information first enters the visual cortex to be interpreted and manipulated by frontal regions (bottom-up processing), mental images take the opposite path, that is, they are initiated by frontal regions and created in the visual cortex (top-down processing). Initial empirical results on this theory are already available, suggesting that aphantasics and non-aphantasics actually differ in the connectivity between several prefrontal regions and the visual occipital network (Milton et al., 2020). This implies an actual absence of visual imagery in aphantasics and not a sole lack of effort. Besides, there is evidence that these differences might be genetically determined since aphantasia occurs particularly often among relatives (Zeman et al., 2020).

While the first larger samples of aphantasics (e.g. Dawes et al., 2020; Zeman et al., 2015, 2020) were assessed with self-report measures such as the *Vividness of Visual Imagery Questionnaire* (VVIQ, Marks, 1973) and the *Questionnaire Upon Mental Imagery* (QMI, Sheehan, 1967), the first behavioural differences have since been observed. For instance, Keogh and Pearson (2018) investigated visual imagery in a sample of aphantasics using a binocular rivalry paradigm and showed that aphantasics, unlike non-aphantasics, were not primed by their own imagery. However, when conceptualizing visual imagery tasks, it should be considered that non-visual alternative strategies could be used, as aphantasics do not necessarily have to differ in their performance from non-aphantasics due to compensatory mechanisms (e.g. Jacobs et al., 2018). Procedures with reaction time measurements turned out to be particularly useful since the non-visual strategy of aphantasics seems to be slower than visual strategies of non-aphantasics (e.g. Crowder, 2018). As Cochrane et al. (2018) showed that visual imagery can be used as a top-down strategy to become more adept in performing visual search tasks, it can be assumed that the visual priming effect found by Keogh and Pearson (2018) would also affect the response time in visual search tasks. Aphantasics should be slower than non-aphantasics. In line with this, Wallace (1988) found that response times in visual search tasks were influenced by vividness of visual imagery, showing that vivid imagers were faster than poor imagers unless poor imagers were taught to use visual imagery as search strategy as well. However, this teaching effect is not expected to occur in aphantasics, as they should not have the possibility of using visual imagery as a search strategy. Hence, if aphantasics do indeed perform worse than non-aphantasics in visual search tasks, this will not only provide further evidence for behavioural differences due to a lack of visual imagery, thus making a further contribution to showing to what extent cognitive differences still elude our knowledge – after all, we assume from the majority of our innate abilities that everyone possesses them (see *everybody-thinks-like-me bias*; Brons, 2019) – it will also underline the importance of visual imagery in attentional guidance for non-aphantasics. Besides, a lack of attentional guidance in aphantasics could have far-reaching consequences for their everyday life, since their real-life visual search could not be facilitated by visual imagery, possibly resulting in worse orientation or goal attainment.

In sum, we aim to show that aphantasics show no or reduced attentional guidance in comparison to non-aphantasics because they cannot use visual imagery. As Kosslyn postulates differential effects of visual imagery on behaviour (while Pylyshyn does not), this effect could be interpreted as evidence for Kosslyn’s theory (e.g. Kosslyn, 1975, 1976, 1978).

## Study 1: Moriya’s Task

Moriya (2018) investigated attentional guidance in visual search tasks in participants with intact visual imagery. They were asked to imagine one of three colours and then to indicate whether one of two presented coloured squares (similar to Landolt rings^1^) was open at the top or bottom. Participants showed longer reaction times in incongruent trials (the distractor had the presented colour) than in neutral trials (neither square had the presented colour) and longer reaction times in neutral trials than in congruent trials (the target had the presented colour). In a second experiment, Moriya (2018) did not instruct participants to visualize the primes, whereby the priming effect disappeared. Since aphantasics are supposed to be unable to perform visualization, the priming effect should also disappear when instructed to visualize (cf. Wallace, 1988). Thus, it is hypothesized that reaction times of non-aphantasics are significantly longer in incongruent than in neutral trials and significantly longer in neutral than in congruent trials, whereas the reaction times of aphantasics should not differ between incongruent, neutral and congruent trials. The same effects will be examined for error rates, although differences in performance are more likely to become apparent in reaction times (cf. Crowder, 2018).

### Method

#### Participants

In order to ensure a sufficiently large sample of aphantasics, participants were recruited via various online forums on the topic of aphantasia as well as via forums on related topics. Non-aphantasics were recruited partly online and partly via the University of Bonn. A total of 2,824 participants completed the study, 207 of whom had to be excluded because they could not reliably guarantee the absence of colour blindness, which could have influenced the performance in Moriya’s Task. Of the 2,617 remaining respondents, 568 stated they were aphantasics (21.7%), 1,324 stated they were non-aphantasics (50.6%), and 725 respondents were not sure about it (27.7%). Participants who indicated that they were unsure whether they had aphantasia or not were excluded from analyses to ensure the validity of the group assignments. Since aphantasics often learn about their condition relatively late in life, the remaining groups were confounded with age (aphantasics: *M* = 30.27, *SD* = 12.84; non-aphantasics: *M* = 25.25, *SD* = 6.50), *t*(693.56) = 8.83, *p* < .001, *d* = 0.44, which could have an impact on reaction times. For this reason, the groups were matched by age according to the procedure of Bacher (2002), creating 531 data pairs. Of the participants, 78.1% indicated that they were male, 19.8% that they were female, and 2.2% that they belonged to a different gender. The age range was 18–69 years (*M* = 27.64, *SD* = 9.05). Most respondents had at least a high school diploma or an equivalent level common in other countries (0.1% no diploma, 5.4% primary school diploma, 11.8% secondary school diploma or equivalent, 39.4% high school diploma or equivalent, 29.3% bachelor’s degree or equivalent, 11.6% master’s degree or equivalent, 2.5% doctoral degree).

#### Visual search task

**Fig. 1:**
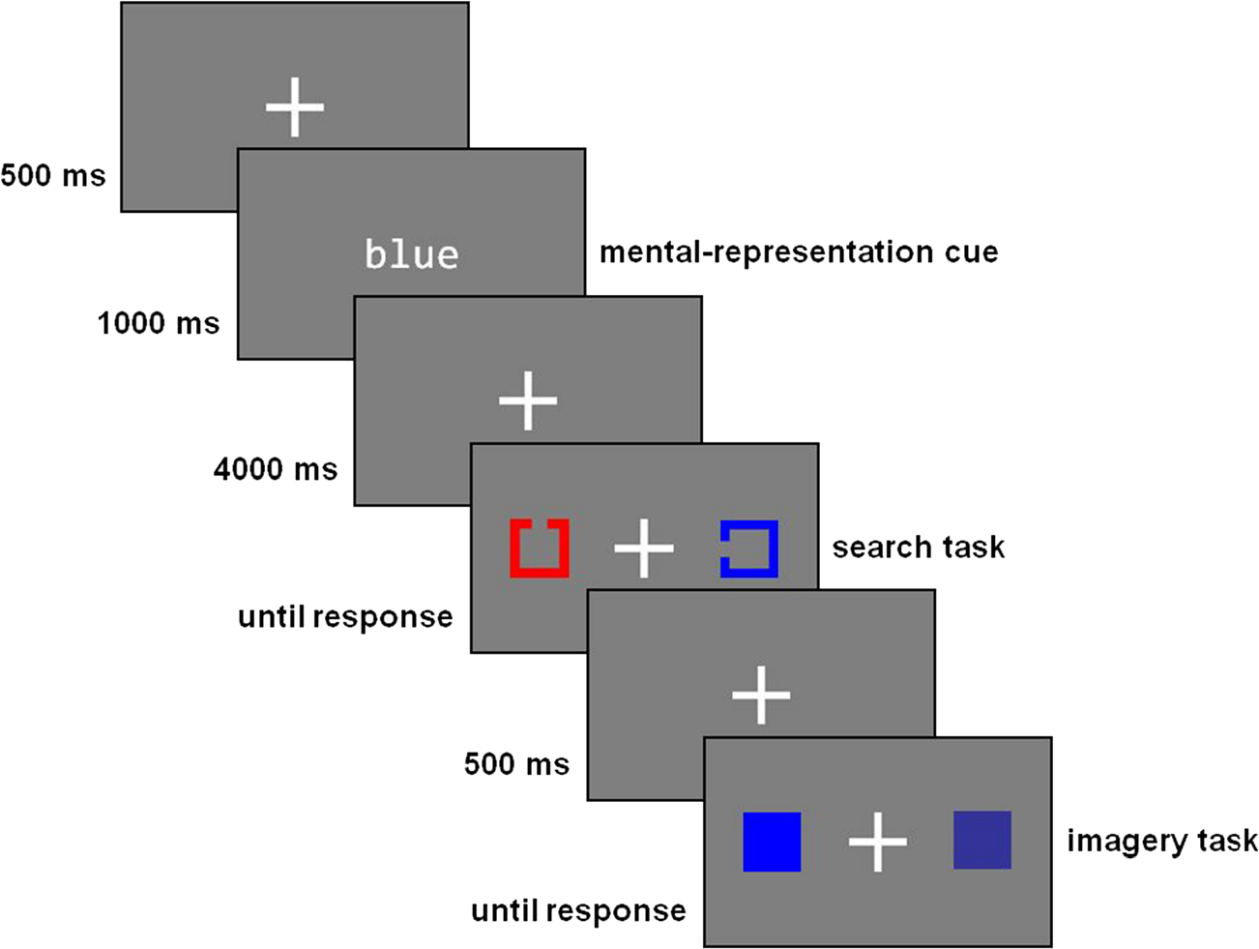
Example of an incongruent trial in Moriya’s Task. Figure adapted based on Moriya (2018)

Moriya’s Task consisted of 72 randomized trials. Participants were instructed to look at a white fixation cross (64 × 64 pixels^2^; RGB: 255, 255, 255) in the middle of a grey screen (RGB: 127, 127, 127). After 500 ms, the name of one of three colours (blue, green or red; 28 pixels per letter [≈ 0.53°]; RGB: 255, 255, 255) appeared for 1,000 ms (mental-representation cue). In the subsequent delay of 4,000 ms, in which the fixation cross was presented again, participants were explicitly asked to visualize the previously presented colour. Subsequently, participants were presented with two coloured outlines of squares (64 × 64 pixels), one of them 350 pixels (≈ 6.54°) to the left and the other 350 pixels to the right of the fixation cross. One of them had an 18-pixel (≈ 0.33°) wide opening either on the top or on the bottom (target). The other outline was open on either the left or the right side (distractor). Participants were asked to indicate whether the target was open at the top or the bottom using the arrow keys (search task). Trials in which the target had the same colour as the mental-image cue are referred to as congruent trials. Trials in which the distractor had the same colour as the mental-image cue are referred to as incongruent trials. Trials in which neither the target nor the distractor had the colour indicated by the mental-image cue are referred to as neutral trials. As in Moriya (2018), the primary colours red (RGB: 255, 0, 0), green (RGB: 0, 255, 0) and blue (RGB: 0, 0, 255) were used. After participants had indicated the position of the opening by means of the arrow keys, the fixation cross was shown for another 500 ms. Afterwards, two coloured squares (64 × 64 pixels) were presented, one 350 pixels to the left and one 350 pixels to the right of the fixation cross. The colours of the squares corresponded to the colour displayed by the mental-image cue in different shades. Red was presented as basic red (RGB: 255, 0, 0), red pigment (RGB: 237, 28, 36), NCS red (RGB: 196, 2, 51) and Munsell red (RGB: 242, 0, 60), green as basic green (RGB: 0, 255, 0), green pigment (RGB: 0, 165, 80), NCS green (RGB: 0, 159, 107) and Munsell green (RGB: 0, 168, 119), and blue as basic blue (RGB: 0, 0, 225), blue pigment (RGB: 51, 51, 153), NCS blue (RGB: 0, 135, 189) and Munsell blue (RGB: 0, 147, 175). Participants were asked to indicate which of the two colour shades most closely reflected the colour they had previously imagined (imagery task). The purpose of this was to encourage participants to visualize more intensively. A graphic representation of Moriya’s Task is shown in Fig. 1.

#### Procedure

The survey was conducted in the period from 12 February 2019 to 14 April 2020 via the online platform psytoolkit.org (Stoet, 2010, 2017), which is comparable to E-prime 3.0 in terms of reliability of response collection (Kim et al., 2019). Since reaction times were first collected locally and only sent back to the PsyToolkit server after the experiment was finished, they were not affected by internet speed. Besides, we instructed the participants to only use computers and allowed only input from real keyboards to at least partially control for display characteristics. However, since devices vary unsystematically between participants, no bias due to display characteristics should be expected. Participation was completely anonymous and participants provided informed consent before beginning the study. The survey was conducted in the English language and in accordance with the World Medial Association Declaration of Helsinki (2013). Before the execution of Moriya’s Task, participants completed two self-report questionnaires, the VVIQ (Marks, 1973) and the *Spontaneous Use of Imagery Scale* (SUIS; Reisberg et al., 2003), to measure subjective visual imagery. The VVIQ (consisting of 16 items using a 5-point Likert scale) assesses the vividness of mental imagery defined as the proximity of imagination to actual perception from *No image at all, you only ‘know’ that you are thinking of the object* to *Perfectly clear and as vivid as normal vision*. The SUIS (consisting of 12 items using a 5-point Likert scale) assesses the frequency of mental imagery from *never appropriate* to *always completely appropriate.* Participants were explicitly instructed to visualize the mental-image cue in Moriya’s Task, followed by three exercise trials and a repetition of the instruction to ensure that participants understood the task. The task took an average of 10 min. At the end of the survey, participants had the opportunity to make open comments. The allocation to the groups of aphantasics and non-aphantasics was based on the participants’ self-assessment. We asked them “Do you have one or more of the following phenomena?” and included the item “Aphantasia (the absence of visual imagination)” on a 4-point Likert scale with the anchors *No, I don’t; Not sure; Yes, self-diagnosed;* and *Yes, diagnosed by an expert*. Since expert-diagnosed aphantasia is still very rare, we decided to assign participants with both self-diagnosed and expert-diagnosed aphantasia to the aphantasia group. Other phenomena inquired about were among others prosopagnosia and synaesthesia, which will be examined in another paper for possible associations with aphantasia.

#### Statistical analyses

Differences in self-reported visual imagery (VVIQ and SUIS) between groups were evaluated by *t*-tests. The analysis of reaction times was done in accordance with Moriya (2018) after the exclusion of incorrect trials. In addition, reaction times that deviated more than three standard deviations from the overall mean were excluded to eliminate reaction times that occurred due to interruptions of the task. Incorrect answers accounted for 5.6% and outliers for 0.3% of all trials. In order to take into account that reaction times often do not have a Gaussian distribution, several generalized linear mixed-effect models (GLMM) were calculated and examined for their model fit (Lo & Andrews, 2015). The model with an inverse Gaussian distribution (raw RT, identity link) had the best model fit and was used to test the effects of group (aphantasics vs. non-aphantasics), priming condition (incongruent vs. neutral vs. congruent trials), and their interaction. To control for variance of random effects, trial numbers and participant identifiers were included. The same analysis was conducted for the error rates using a Gaussian distribution (identity link). Denominator degrees of freedom were estimated using Satterthwaite’s method (Kuznetsova et al., 2017). If an omnibus test was significant, sequential post hoc tests with adjusted *p* values were calculated.

### Results

#### Validation of self-classification

The difference in self-reported visual imagery was significant: Aphantasics showed worse visual imagery than non-aphantasics, both in the VVIQ (aphantasics: *M* = 20.10, *SD* = 6.92; non-aphantasics: *M* = 65.84, *SD* = 11.48), *t*(872.67) = 78.90, *p* < .001, *d* = 4.84, and in the SUIS (aphantasics: *M* = 17.94, *SD* = 6.00; non-aphantasics: *M* = 39.16, *SD* = 9.02), *t*(922.44) = 45.16, *p* < .001, *d* = 2.77.

#### Reaction times

**Fig. 2:**
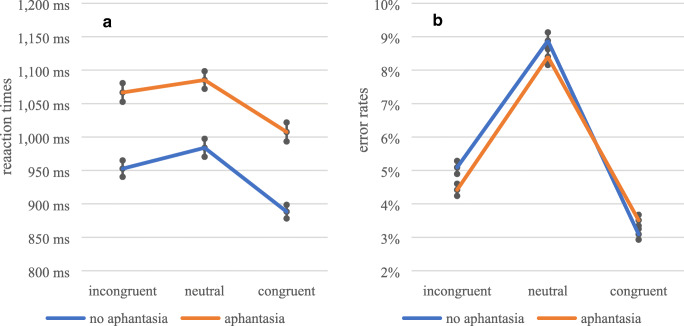
Reaction times (**A**) and error rates (**B**) in Moriya’s Task, dependent on group and priming condition. Depicted are means ± 1 SEM

The GLMM revealed a significant main effect of priming condition, *F*(2, 259.49) = 3.61, *p* = .028, ω_p_^2^ = .02. While participants were as fast in incongruent trials (*M* = 1,034.92 ms, *SE* = 9.50 ms) as in neutral trials (*M* = 1,039.95 ms, *SE* = 9.09 ms), *z* = 0.79, *p* = .661, participants were significantly faster in congruent trials (*M* = 950.62 ms, *SE* = 8.45 ms) than in neutral trials, *z* = 2.27, *p* = .045, *d* = 0.14. Furthermore, aphantasics (*M* = 1,066.90 ms, *SE* = 7.82 ms) were significantly slower than non-aphantasics (*M* = 950.21 ms, *SE* = 6.88 ms), *F*(1, 222.88) = 4.67, *p* = .032, ω_p_^2^ = .02. However, no interaction effect between priming condition and group was found, *F*(2, 283.99) = 0.37, *p* = .693. The reaction times dependent on group and priming condition are shown in Fig. 2A.

#### Error rates

The GLMM revealed a significant main effect of priming condition on error rates, *F*(2, 225.24) = 76.03, *p* < .001, ω_p_^2^ = .40. Participants made fewer errors in incongruent trials (*M* = 4.8%, *SE* = 0.1%) than in neutral trials (*M* = 8.6%, *SE* = 0.2%), *z* = 8.57, *p* < .001, *d* = .55, and more errors in neutral trials than in congruent trials (*M* = 3.5%, *SE* = 0.1%), *z* = 7.88, *p* < .001, *d* = .50. Since participants were faster in congruent than in neutral trials and made simultaneously fewer errors, a speed-accuracy trade-off cannot be assumed. The interaction effect between priming condition and group, *F*(2, 236.03) = 0.68, *p* = .505, as well as the main effect of group, *F*(1, 957.39) = 0.12, *p* = .727, were not significant. Error rates dependent on group and priming condition are shown in Fig. 2B.

### Discussion

Participants were faster and made fewer errors in congruent trials than in neutral trials, thereby partly replicating the priming effect of Moriya (2018). Unexpectedly, however, participants also made fewer errors in incongruent trials than in neutral trials and were as fast in incongruent trials as in neutral trials, a result not found by Moriya (2018). This pattern could have reflected a predominant strategy of searching the primed colour before responding to the actual task, leading to a higher response time when the colour was not present – neither as part of the target nor as part of the distractor – and the participants had to realize this first. This effect might not have been found by Moriya (2018) due to the small sample size in that study. Furthermore, contrary to the hypothesis, no interaction effect between group and priming condition was found. This finding suggests that aphantasics can be primed in the same way as non-aphantasics. However, two limitations have to be considered with regard to this interpretation.

First, it is conceivable that at least the (unconscious) colour imagery of aphantasics is unimpaired, since it is based on its own distinct brain region, namely the V4 (Bartels & Zeki, 2000). For example, Jacobs et al. (2018) found no differences in change-detection accuracy between aphantasics and non-aphantasics when using colours or simple shapes. Therefore, another visual search task should be designed using objects differing in many features instead of mere colours as primes.

Second, 214 participants of the total sample complained about the length and complexity of Moriya’s Task. Participants did not only have to visualize the cue, but also had to select an answer based on a different feature, namely the gap in the outline of the Landolt square, whereas the answer did not correspond to the position of the target, but to the position of the gap. Afterwards, they had additionally to choose the colour corresponding to their visualization in the imagery task. This resulted in a 65.0% dropout rate (only 2,824 of 8,075 initial participants completed the task). It is therefore possible that, to simplify the task, the remaining non-aphantasics did not engage in visualizing the prime, resulting in acting like aphantasics and not showing any differences in comparison to them. This assumption is supported by the fact that non-aphantasics (34.7%) gave randomly distributed answers in the final imagery task as often as aphantasics (36.9%), χ^2^(1) = 0.60, *p* = .274, although, according to Mannaert et al. (2017), when visualizing a colour, imagers tend to be biased towards visualizing the same colour over and over again (cf. Moriya, 2018). In comparison, in Moriya (2018), non-aphantasics gave randomly distributed answers only in 12.3% of the cases. The main effect of the priming condition in the present study could therefore be due to non-visual (e.g. semantic) priming in both aphantasics and non-aphantasics rather than due to visual imagery priming taking place only in non-aphantasics. Related to this is the fact that the processing of the prime in Moriya’s Task was actually not necessary to select the correct response as the correct response only depended on features of the target and not on features of the prime. Therefore, both groups may have neglected explicit processing of the prime, ultimately reducing differences between them. Instead, both groups might have made a priori representations of the search display or the target because only characteristics of the target (a gap either on the top or bottom) were necessary to identify the target and select the right answer as fast as possible. This might also explain why non-aphantasics were generally faster than aphantasics. Specifically, while non-aphantasics could prepare themselves with visual representations, aphantasics had to use non-visual representations, which would be less effective because cross-modal priming tends to be less effective than intra-model priming (see *modality effect*, Richardson-Klavehn & Bjork, 1988). Besides, non-aphantasics could benefit from intersensory facilitation, an effect that can speed up reaction times, when two representations (e.g. visual and non-visual) appear concurrently (Colonius & Diederich, 2012). Another explanation for the main effect of group could be the higher switch costs for aphantasics when trying to switch between visualization and visual search, as the process of visualization should be automatic in non-aphantasics while being extremely difficult or impossible for aphantasics (see Schneider & Anderson, 2010). In contrast, a main effect for error rates might not have been evident due to the simplicity of the actual search task (error rates of about 5.6%, which equals roughly four out of 72 trials).

## Study 2: Spontaneous use of visual imagery visual search task

Major points of criticism regarding the use of Moriya’s Task to find differences between aphantasics and non-aphantasics were the dependence on pure single-feature colour imagery, the complexity of the task depleting the capacity for visual imagery, and the non-necessary explicit processing of the prime. For this reason, the *Spontaneous Use of Visual Imagery Visual Search Task* (SUVI Task) was developed, which is also based on priming through visual imagery, but instead of colours whole objects have to be visualized. The complexity of the task was also reduced by no longer making the selection of the target dependent on attributes of the target but on the target itself. After presenting a verbal cue, either two words (word trials) or two pictures (image trials) are presented, one of which corresponds to the cue and has to be selected (target). Therefore, the cue has to be processed explicitly. Petilli et al. (2020) showed that visual properties of objects denoted by verbal cues are automatically activated and influence the processing of target words even when visual processing is neither solicited nor required, and Amit et al. (2017) showed on a neuronal level that participants create visual representations even when the information actually has to be processed verbally (although the participants themselves do not necessarily have to be aware of these images; Koenig-Robert & Pearson, 2019). Thus, it can be assumed that non-aphantasics actually visualize the prime. On the other hand, aphantasics, by definition, cannot create visual representations, which should lead to slower reaction times in image trials compared to non-aphantasics. This is the case regardless of the aphantasics’ ability to be primed non-visually (e.g. semantically) because intra-model priming (Richardson-Klavehn & Bjork, 1988) and combined visual and non-visual priming (Colonius & Diederich, 2012) in non-aphantasics should be stronger than only cross-modal non-visual priming in aphantasics. However, in word trials, the reaction times should not differ between groups as both groups are equally able to be primed non-visually, and visual imagery seems not to influence pure visual word recognition (Allen et al., 1994). Therefore, as in Study 1, an interaction effect between trial condition and group was expected. A main effect of trial condition will be reported but not interpreted in terms of mental imagery since the target stimuli in both conditions were too different and it is likely that the performances in both conditions differ due to perceptual processes rather than due to imagery. Perceptual processing of pictures is found to be faster than the perceptual processing of words (Pellegrino et al., 1977).

### Method

#### Participants

The sample consisted of a sub-sample of the 2,591 participants from Study 1 who agreed to also participate in Study 2. Since in Study 2 the primes consisted of words and not pictures, all non-native English speakers had to be excluded, as the open comment section showed that non-native English speakers mentally translated the words, which may have led to increased verbal processing interfering with the expected effect. Of the remaining 1,536 respondents, 355 stated they were aphantasics (23.2%), 742 stated they were non-aphantasics (48.3%), and 439 respondents were not sure (28.6%). As in Study 1, participants who indicated that they were unsure whether they had aphantasia or not were excluded from analyses to ensure the validity of group assignments. Again, the remaining groups were matched by age according to the procedure of Bacher (2002), creating 325 data pairs. Of the participants, 75.8% indicated that they were male, 20.6% that they were female, and 3.5% that they belonged to a different gender. The age range was 18–68 years (*M* = 28.74, *SD* = 9.64). Most of the respondents had at least a high school diploma or an equivalent level common in other countries (0.2% no diploma, 7.1% primary school diploma, 10.6% secondary school diploma or equivalent, 38.2% high school diploma or equivalent, 30.6% bachelor’s degree or equivalent, 10.6% master’s degree or equivalent, 2.8% doctoral degree).

#### Visual search task

**Fig. 3:**
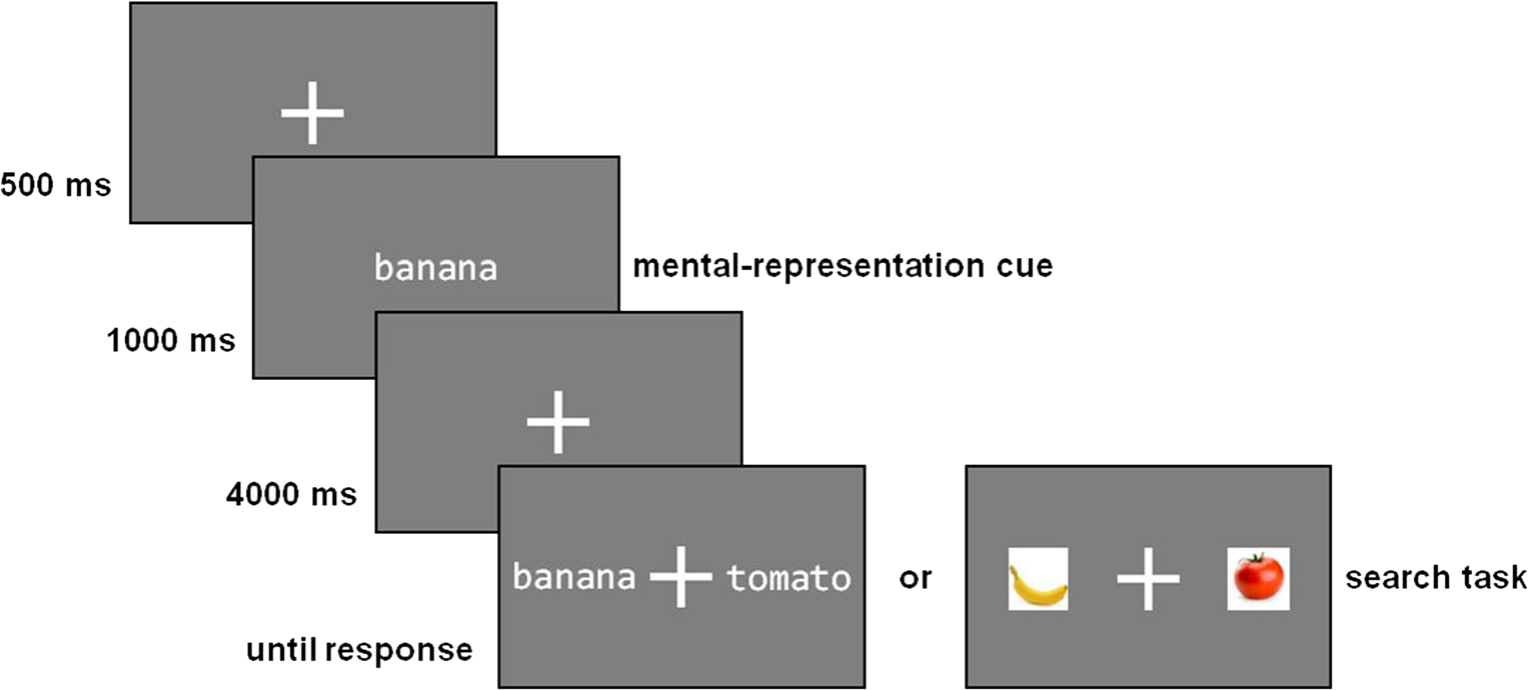
Example of a word and an image trial in the Spontaneous Use of Visual Imagery Visual Search Task (SUVI Task)

A white fixation cross (64 × 64 pixels^3^; RGB: 255, 255, 255) was presented on a grey screen (RGB: 127, 127, 127) for 500 ms before one of four words (banana, cucumber, pumpkin or tomato; 28 pixels per letter [≈ 0.53°]; RGB: 255, 255, 255) was presented for 1,000 ms (mental-representation cue). Then, the fixation cross appeared again for 4,000 ms during which participants were asked to visualize the cue. Subsequently, participants were presented with either two words (28 pixels per letter [≈ 0.53°]; RGB: 255, 255, 255) or two images (150 × 150 pixels [≈ 2.81°]), one of them 350 pixels (≈ 6.54°) to the left and the other 350 pixels to the right of the fixation cross. Participants were asked to use the arrow keys to indicate which of the two stimuli corresponded to the mental-representation cue (target), i.e. either depicted the same word (word trials) or an image of the object (image trials) represented by the mental-representation cue (search task). The distractor each time represented one of the cues not used in that trial. Twenty-four word and 24 image trials were presented. A graphic representation of the SUVI Task is shown in Fig. 3.

#### Procedure

The SUVI Task was conducted via the online platform psytoolkit.org (Stoet, 2010, 2017), as with Study 1. Similar to first study, after three exercise trials, the instructions were repeated before starting the main part of the task in which reaction times and error rates were recorded. As planned, the task was shorter than Moriya's task and took an average of 5 min.

#### Statistical analyses

Differences in self-reported visual imagery (VVIQ and SUIS) between groups were evaluated by *t*-tests. With regard to the SUVI Task, the same trial exclusion procedure as in Study 1 was applied. Incorrect answers accounted for 4.9% and outliers for 1.1% of all trials. Again, the GLMM with the Inverse Gaussian distribution (raw RT, identity link) had the best model fit and was used to test the effects of group (aphantasics vs. non-aphantasics), trial condition (word vs. image trials), and their interaction. To control for random effects variance, trial numbers and participant identifiers were included. The same analysis was conducted for the error rates using a Gaussian distribution (identity link). Denominator degrees of freedom were estimated using Satterthwaite’s method (Kuznetsova et al., 2017).

Since the interaction effect of group and trial condition was significant for reaction times, a priming score was computed (reaction times in word trials minus reaction times in image trials), thereby controlling for the base reaction time of individual participants. High difference scores are thought to reflect high vividness of visual imagery, since non-aphantasics should react faster in image trials than aphantasics. To validate the priming score, additional correlation analyses with the scores of VVIQ and SUIS as well as an additional group comparison between aphantasics and non-aphantasics were conducted.

### Results

#### Validation of self-classification

The difference in self-reported visual imagery was significant: Aphantasics showed worse visual imagery than non-aphantasics, both in the VVIQ (aphantasics: *M* = 19.84, *SD* = 6.32; non-aphantasics: *M* = 66.30, *SD* = 10.81), *t*(522.02) = 66.88, *p* < .001, *d* = 5.24, and in the SUIS (aphantasics: *M* = 17.46, *SD* = 5.50; non-aphantasics: *M* = 39.54, *SD* = 8.78), *t*(544.23) = 38.42, *p* < .001, *d* = 3.01.

#### Reaction times

**Fig. 4:**
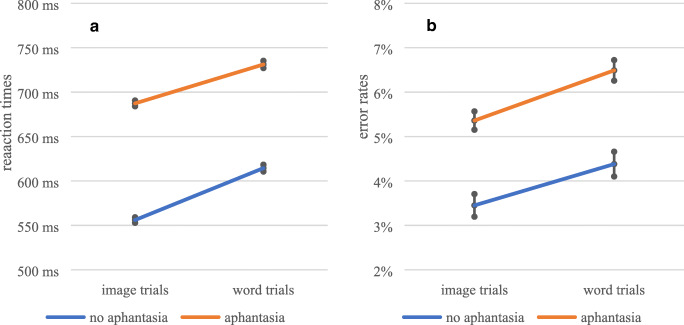
Reaction times (**A**) and error rates (**B**) in the Spontaneous Use of Visual Imagery Visual Search Task, dependent on group and priming condition. Depicted are means ± 1 SEM

The GLMM revealed a significant main effect of trial condition, *F*(1, 138.67) = 496.18, *p* < .001, ω_p_^2^ = .78, as well as a significant main effect of group, *F*(1, 514.41) = 7.45, *p* = .007, ω_p_^2^ = .01. Participants were slower in word trials (*M* = 709.22 ms, *SE* = 2.62 ms) than in image trials (*M* = 585.19 ms, *SE* = 2.50 ms), and aphantasics (*M* = 672.78 ms, *SE* = 2.85 ms) were slower than non-aphantasics (*M* = 621.62 ms, *SE* = 2.32 ms). The interaction effect between trial condition and group was significant, *F*(1, 150.37) = 5.54, *p* = .020, ω_p_^2^ = .03. Post hoc *t*-tests revealed significant differences between aphantasics and non-aphantasics in image trials, *t*(570.28) = 4.02, *p* < .001, *d* = 0.25, but not in word trials, *t*(185.88) = 1.19, *p* = .236. In order to investigate whether the effect was only significant due to the large sample size, sequential Bayesian analyses were calculated, showing that these results can be found independent of sample size (see Figs. S1 and S2, Online Supplementary Material). The reaction times dependent on group and trial condition are shown in Fig. 4A.

Additional correlation analyses of the priming score (reaction times in word trials minus reaction times in image trials) with the VVIQ, *r* = .10, *p* = .006, and the SUIS, *r* = .09, *p =* .014, were significant. Moreover, aphantasics (*M* = 119.67 ms, *SE* = 5.62 ms) showed lower priming scores than non-aphantasics (*M* = 133.03, *SE* = 4.42), *t*(613.83) = 1.87, *p* = .031, *d* = 0.15.

#### Error rates

The GLMM revealed a significant main effect of trial condition, *F*(1, 65.51) = 47.69, *p* < .001, ω_p_^2^ = .41, but no significant main effect of group, *F*(1, 602.68) = 5.07, *p* = .055. Participants made more errors in word trials (*M* = 5.9%, *SE* = 0.2%) than in image trials (*M* = 3.9%, *SE* = 0.2%). Since the participants were slower in word trials and made simultaneously more errors, a speed-accuracy trade-off cannot be assumed. However, since the overall error rate was lower than in Moriya’s Task, while the reaction times were simultaneously faster, the assumption of the SUVI Task being less complex than Moriya’s Task seems to be met. An interaction effect between trial condition and group, *F*(1, 363.37) = 0.13, *p* = .717, was not significant. The error rates dependent on group and trial condition are shown in Fig. 4B.

### Discussion

A main effect of trial condition was significant for reaction times and error rates, confirming Pellegrino et al. (1977), who found that perceptual processing of pictures is faster than perceptual processing of words. Since this effect is mainly based on perceptual processes, no conclusion about visual imagery in aphantasics and non-aphantasics can be made. However, an interaction effect between group and trial condition was significant, suggesting that aphantasics cannot be primed by their own imagery in contrast to non-aphantasics. Non-aphantasics reacted faster to images than aphantasics, whereas no such effect was found for words. This implies that non-aphantasics use visual imagery as a strategy to select the target as fast as possible, while aphantasics have to rely solely on non-visual strategies. Furthermore, the resulting priming score, which should indicate the degree of visual strategy usage, could be validated using the already established subjective measures of visual imagery VVIQ and SUIS.

Regarding the error rates, no interaction or main effect could be found, probably because of a floor effect due to the simplicity of the task (error rates of about 4.9%, which equals roughly two out of 48 trials). Instead, a group main effect was found for reaction times, indicating that the non-visual strategy of aphantasics is slower (cf. Crowder, 2018) than the combined visual and non-visual strategy of non-aphantasics, probably due to higher flexibility of the latter. For example, non-aphantasics could additionally compare visual representations of the cue (i.e. pictures of the letters) with the target in the word trials, while aphantasics had to rely completely on processing these words non-visually. However, since an interaction effect was found and post hoc tests between aphantasics and non-aphantasics were not significant in word trials, the group main effect should not be given too much importance.

## General discussion

To the best of our knowledge, the examined sample is the largest sample of aphantasics that has ever been experimentally tested for visual imagery. The main effect of priming condition for both reaction times and error rates in Study 1 suggests that aphantasics were primed in the same way as non-aphantasics, although this might be due to task characteristics of Moriya’s Task.^4^ Mere colour imagery may have led to visual priming in both groups or, more likely, the missing necessity to process primes visually, while simultaneously being confronted with overly complicated instructions may have led to purely non-visual priming in both groups. These limitations were addressed in the SUVI Task, in which an interaction effect between group and trial condition was found. Non-aphantasics were faster in image trials than aphantasics, probably due to visual imagery, while showing the same priming effects in word trials.

Since it could be shown that priming in image trials is weaker in aphantasics than in non-aphantasics, this leads to the conclusion that aphantasics lack attentional guidance through visual imagery. Moreover, it has to be mentioned that aphantasics do not lose attentional guidance completely, probably due to compensatory cross-modal non-visual priming processes, which, however, tend to be weaker than intra-modal priming (Richardson-Klavehn & Bjork, 1988) or priming with combined modalities (Colonius & Diederich, 2012). On the other hand, it could be shown that visual imagery enhances attentional guidance when visual imagery is unimpaired.

## Limitations

The first limitation of the present studies appears to be the lack of validity in assigning participants to the groups, as this was based on self-assessment, which may have been impaired due to a lack of introspection (Schwitzgebel, 2002). This may have resulted in some aphantasics being classified as non-aphantasics and some non-aphantasics being classified as aphantasics, which may ultimately have led to a reduction of the effect. Furthermore, it is possible that aphantasia can be distinguished into two further forms: One in which no mental images are actually produced, which also means that no priming occurs (Keogh & Pearson, 2018), and one in which mental images are produced but remain unconscious (Kwok et al., 2019) and still lead to priming effects. In our studies, both forms would probably have been assigned to the aphantasics group, which, again, would have reduced the validity of the assignment to the groups. However, such a misclassification would only render our results more conservative as it would decrease an otherwise bigger effect. Nevertheless, in subsequent studies, the classification of the participants into aphantasics and non-aphantasics should be based on objective criteria, for example on the binocular rivalry paradigm from Keogh and Pearson (2018), or on brain activation during imagery tasks (e.g. Cui et al., 2007). Yet, neither validation criteria were applicable in our large-scale online studies, and MRI or lab experiments demand a huge pre-screening in order to obtain a sufficient sample size to warrant statistical power.

Second, it can be claimed that in our study it was not possible to control whether non-aphantasics really visualized the primes. This is an important point of criticism that affects imagination research in general. However, given that we found behavioural differences in two samples that primarily differ in their ability to visualize, it can be assumed that these behavioural differences are due to different cognitive processes while performing the task. In addition, non-aphantasics tend to create visual representations of verbal cues even when information is actually required to be processed verbally (Amit et al., 2017; Petilli et al., 2020), as was the case in the SUVI Task. Participants needed to explicitly process the cue to be able to perform the visual search task and they were provided with simple instructions to ensure that they had enough capacity to visualize (as seen in the faster reaction times in the SUVI Task in comparison to Moriya’s Task, while showing the same overall error rate). Further evidence that imagery was actually performed would require much more comprehensive methods than behavioural tasks (e.g. fMRI), which would go beyond the scope of this paper.

Third, despite the previous evidence that aphantasics actually exist (e.g. Milton et al., 2020), it can be argued that we could not control if aphantasics really could not visualize the primes. They could have intentionally or unintentionally suppressed their visual imagery or just told us that they had no imagery. However, there is no obvious reason why aphantasics should fake an absence of visual imagery and it would almost be impossible to fake reaction times in the millisecond range. Furthermore, it is not clear if suppression of visual imagery is even possible as visual processing seems to be automated (Amit et al., 2017; Petilli et al., 2020). Even if unintentional suppression of visual imagery was possible – for example because the participants assumed they cannot use it anyway – this would be functionally equivalent to a loss of visual imagination as it would impact the information processing of aphantasics not only during the task but also during the remaining aspects of their lives.

Finally, we need to consider if there is another possible interpretation of the interaction effect as aphantasia is congenital and the assignment to the groups is therefore quasi-experimental. Thus, besides a lack of attentional guidance, differences in pure object identification could have also influenced the reaction times. While this cannot be ruled out completely by our data, as no distinction between visual search and object identification was made, it is conceivable that such object identification effects might also be due to attentional guidance because targets and their features could also be visualized in advance. Furthermore, object classification effects should not play a major role in the SUVI Task, as the same four pictures were repeated many times and new pictures did not have to be classified into a category first. However, there are some other alternative explanations for differences in the visual search task performance between aphantasics and non-aphantasics, for example more conservative decisions when images are involved or impaired associations between pictures and words, but all of these differences would ultimately be caused by the absence of visual imagination in aphantasics. Therefore, until these processes can be distinguished within aphantasics, it seems most obvious to look for the cause of the present results in attentional guidance, since the effect of visual imagery on attentional guidance has been shown previously (Moriya, 2018).

## Implications

Regardless of the exact mechanisms resulting in aphantasics being slower in image trials than non-aphantasics, priming through visual imagery could be used in the future to distinguish between aphantasics and non-aphantasics independently of self-reports (e.g. Zeman et al., 2015, 2020), as self-report measures are rather unreliable due to the constraints of introspection (Schwitzgebel, 2002). The large uncertainty regarding self-assessment of one’s own imagery abilities is also reflected in the fact that 27.7% and 28.6%, respectively, of the participants in our studies were not able to classify themselves as aphantasics or non-aphantasics with certainty. However, the priming effect is currently too small and too unreliable to be yet used as a classification tool. A more valid group assignment (e.g. via fMRI) and suppression of non-visual compensatory strategies in a dual-task design may strengthen the effect in the future.

Nevertheless, the present study provides new evidence for Kosslyn’s (1981, 2005) theory of image-dependent processing since behavioural differences between aphantasics and non-aphantasics could be found. Since Pylyshyn (1973, 2002, 2003) considers mental imagery to be merely an epiphenomenon of propositional processing, the absence of such imagery in aphantasics should not have produced behavioural differences between aphantasics and non-aphantasics. In the future, it should also be investigated whether the lack of attentional guidance through visual imagery can influence how aphantasics perceive their environment and therefore hinder achievement of their goals in real-life settings of visual search.

## Conclusion

The present study is the first to reveal behavioural effects of aphantasia in a large sample of aphantasics. While non-aphantasics are influenced in their performance in visual imagery tasks by their visual imagery, aphantasics are not. This is particularly interesting since participants were explicitly asked to visualize the primes, and Wallace (1988) showed that even poor imagers can be encouraged to use visual imagery strategies in visual search tasks. Thus, it can be assumed that the absence of visual imagery in aphantasics is a qualitative rather than a quantitative phenomenon and that it requires more effort (e.g. drug-induced imagery; dos Santos et al., 2018) to restore visual imagery in aphantasics than in poor imagers. Attentional guidance in aphantasics seems to be fundamentally impaired.

## Supplementary Information

ESM 1 (DOCX 311 kb)

## Footnotes

1 We thank Dr. Moriya for providing the original stimuli, which allowed us to be able to keep the replication as close to the original as possible.

2 The task was presented in a resolution of 1,920 × 1,080 pixels. Assuming an average monitor size of 17 in. and an average viewing distance of 60 cm, the stimulus size corresponds to a visual angle of 1.19° × 1.19°. The same dimensions are used for the further calculation of visual angles. However, since the study was conducted online, the exact dimensions may vary between participants.

3 The task was presented in a resolution of 1,920 × 1,080 pixels. Assuming an average monitor size of 17 in. and an average viewing distance of 60 cm, the stimulus size corresponds to a visual angle of 1.19° × 1.19°. The same dimensions are used for the further calculation of visual angles. However, since the study was conducted online, the exact dimensions may vary between participants.

4 To exclude the possibility of different results due to different sample characteristics, we repeated the analyses of Study 1 with the sample of Study 2. All effects persisted with exception of the main group effect for reaction times, which was not significant anymore. However, it was barely significant in the original sample and not part of our theoretical framework anyway.
