# Supplementary material for: Imagine, and you will find – Lack of attentional guidance through visual imagery in aphantasics
Source: Atten Percept Psychophys. 2021 Apr 20;83(6):2486–97. doi: 10.3758/s13414-021-02307-z (PMC8302533; doi:10.3758/s13414-021-02307-z)
Supplement: Supplementary file 1 — (DOCX 311 kb) [file 13414_2021_2307_MOESM1_ESM.docx]

**Supplemental Material**


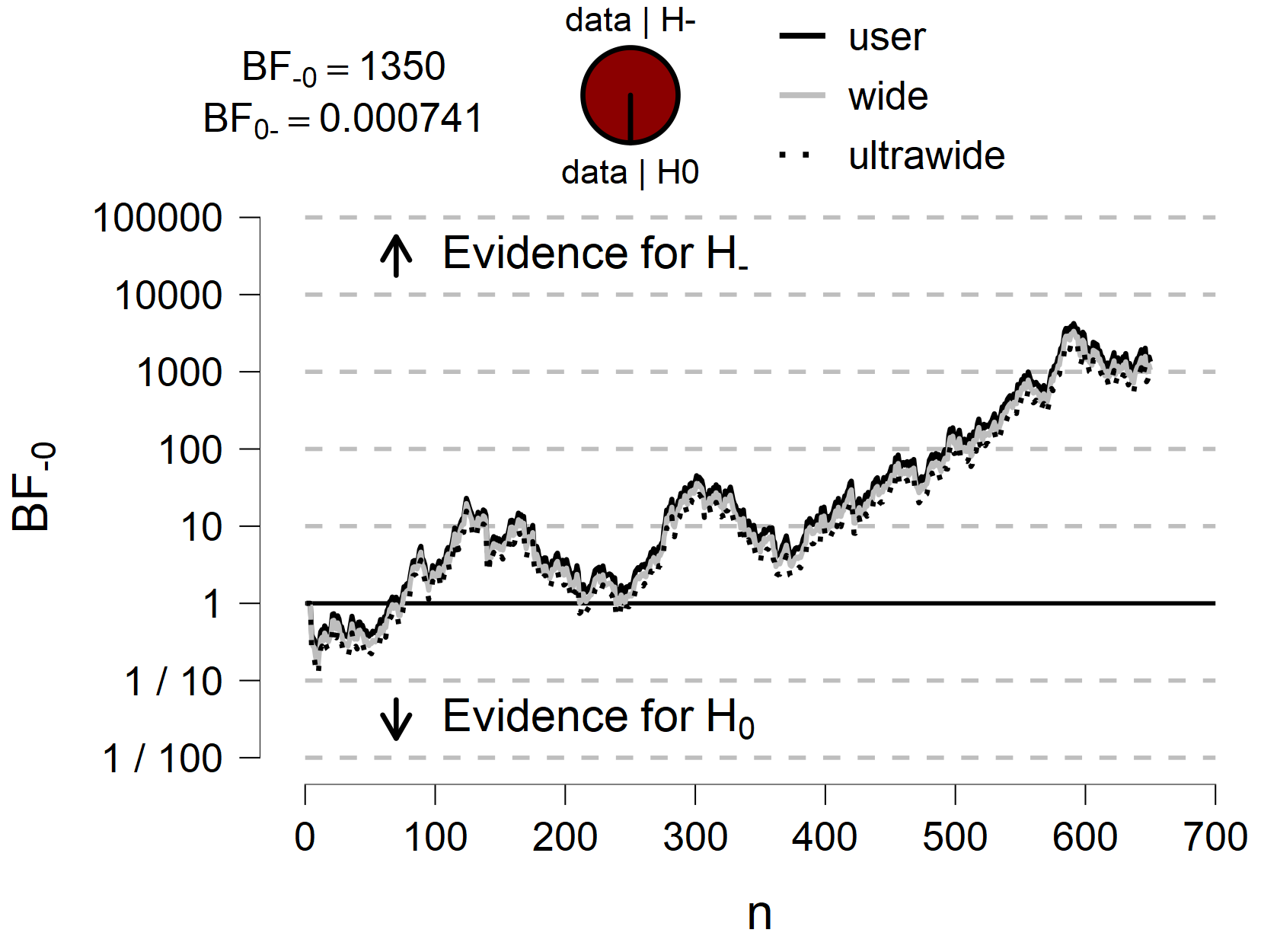


*Fig. S1.* Sequential Bayesian analysis of the reaction time differences between aphantasics and non-aphantasics in image trials (JASP, Version 0.14).


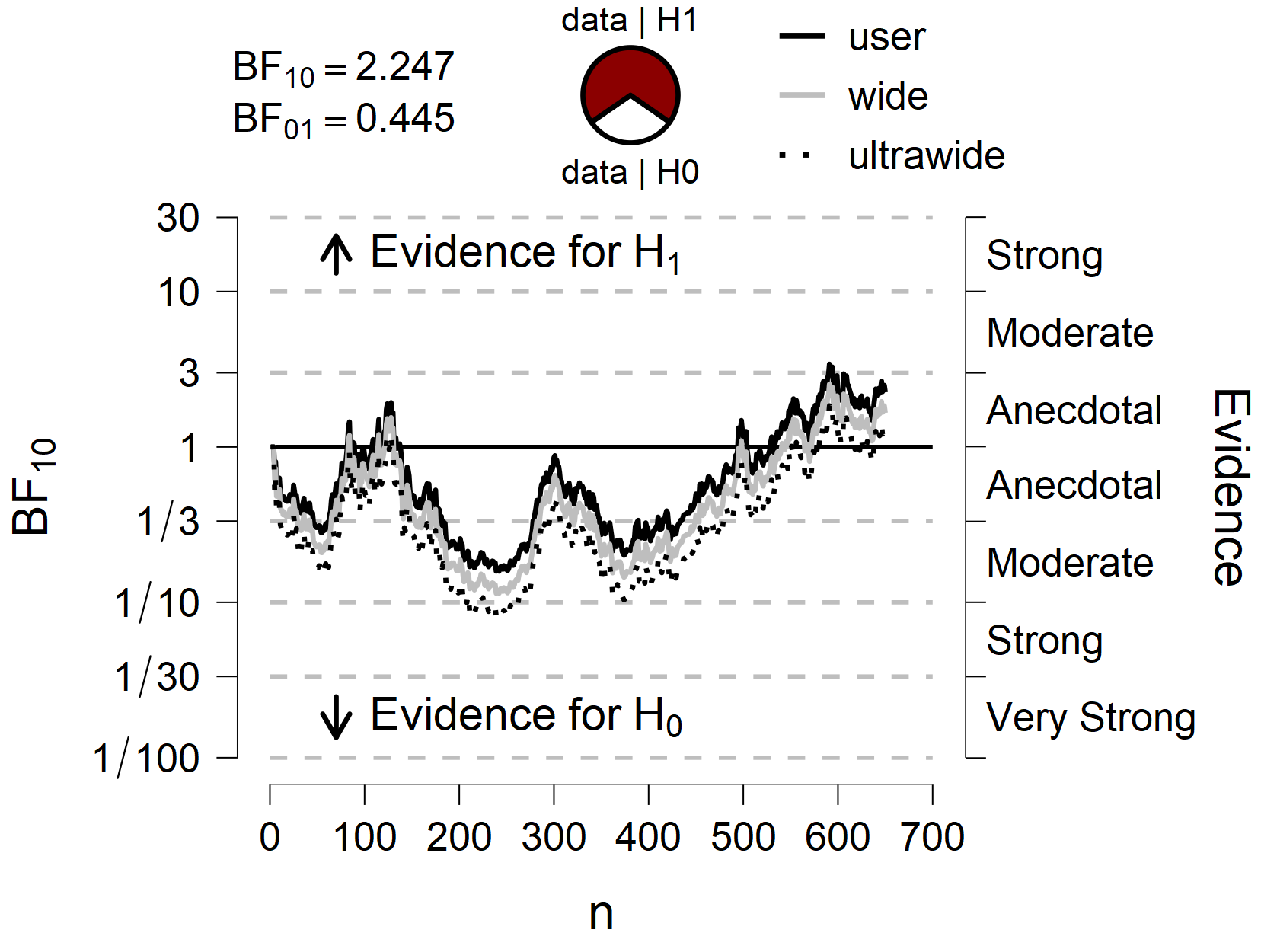


*Fig. S2.* Sequential Bayesian analysis of the reaction time differences between aphantasics and non-aphantasics in word trials (JASP, Version 0.14).
